# Remarks on the Malignant Intermittent and Remittent Fevers *of Limestone County, Alabama*

**Published:** 1839-11-09

**Authors:** T. Stith Malone

**Affiliations:** Athens, Alabama


### REMARKS ON THE MALIGNANT INTERMITTENT AND REMITTENT FEVERS *of Limestone County, Alabama.* By T. STITH MALONE, M. D.

To the Editors of the Medical Examiner.

GENTLEMEN,—I am induced to offer you a few remarks, elicited by the perusal of Nos. 34 and 38 of your very interesting *weekly*,—more particularly, the letter of Dr. J. R. Buck, of Memphis, Tennessee. A close practice of six years in Limestone county, Alabama, has given me some experience in the diseases to which I wish to call your attention. Limestone county is watered by the Tennessee and Elk rivers, and some eight or ten smaller streams, with a number of *mill dams* upon several of the latter. Hence, there are very sufficient causes, especially in dry seasons, to originate the most violent grades of intermittent and remittent fevers. So violent is the character of these fevers, that it is no unusual circumstance for patients to die in the first chill. It is more common, however, for the first, and, occasionally, the second, to be light, confining the patient to bed but for a few hours; the second or third, if no means be used to check the attack, will prostrate him, when he will have a cold skin, intense thirst, great oppression of the chest, with stricture across it, producing fainting, and an intolerable sensation of suffocation, with severe pain, upon every attempt at respiration. There is occasionally great pain in the epigastric region, with jactitation, and a general feeling of numbness or deadness. The pulse is very uncertain: I have seen it unaltered, for forty-eight or sixty hours, in frequency varying in different individuals from forty-four to one hundred and thirty-eight, or one hundred and forty, occasionally falling in the number of beats as the cold stage goes off, though more generally remaining the same, or increasing. As to its volume, etc. etc., I have seen almost every variety. In young subjects, epileptical attacks, in from four to eight hours after the induction of the chill, are sometimes seen.

The success of the prevailing plan of treatment in this county, is decidedly more gratifying than seems to be the case in the districts of some of your correspondents. The age of the patient, his idiosyncracies, etc., not being unfavourable, if the physician be called at the commencement of the attack, he expects to lose but few patients, say, not more than two out of fifteen cases,—and if there be no relapses, or improprieties on the part of the patient, even much less.

I give you my general course of treatment, which I believe is pretty generally that of the physicians of the county. Called to a case of the character to which I have alluded, I should at once tie up both arms, and open a vein in each, to relieve the engorged and congested condition of the lungs. We sometimes see the oppression in the chest, which appears to be concentrated in a single sharp pain, that is for a moment most agonizing, at once give way, leaving the patient partially, if not entirely relieved of his feelings of suffocation. The change in the character of the attack about the chest, begins when from two to six ounces of blood have been abstracted; and though occasionally alarmed by its acuteness, I have never seen it fail to give way, if the abstraction of blood be persevered in. As soon as the chill is over, we give powders of

Calomel gr. v.
Nitrate of potash gr. vi.
Ipecac. ½ gr.
Every two or three hours, till four or five doses have been taken.

If there be irritable stomach, or a sensation of weight or oppression there, we cup the epigastrium to the amount of eight or twelve ounces—then apply stimulating warm poultices, to be followed or not, as occasion may require, with mustard or fly blisters. If the stomach be irritable, the above powders, with acetate of ammonia, in *cold* water, every hour or two, may be given; if not, and the parched mouth, and dry tongue, and restlessness do not relax, a quarter of a grain of tartar emetic should be added to the powders. This course, in my estimation, reduces the congestion, and brings about a general diffusion of the energies, an equalized circulation. We then throw in large doses of quinine, to keep up the restored balance of the circulation, and to prevent the reaccumulation of blood in the large vessels, the lungs, or the stomach, liver, and spleen. I make it a rule to commence the exhibition of quinine *at least* eight hours before the expected return of the chill; and my experience is, that though the mouth be dry, the skin hot, etc., if you precede the quinine with sufficient depletion, and then give doses of it large enough, (say fifteen to twenty-five grains, every two to four hours,) the tongue will become moist, the skin will relax, and "great drops of sweat will come rolling out." If, by the most active and energetic depletion, which is much assisted by the free exhibition of cold iced water, I can quiet the patient sufficiently, (I do not know how else to express it, as the pulse is a very uncertain test, and the skin may be cool or the contrary,) I commence with the quinine, and get the patient's system under its influence as early as possible after the first chill. Of the cases of relapse, I shall merely remark that they are exceedingly troublesome, but nearly always recover under the harassing effects of salivation.

*Athens*, *Alabama*, October 23d, 1839.

